# A Case Report: Can Citrin Deficiency Lead to Hepatocellular Carcinoma in Children?

**DOI:** 10.3389/fped.2019.00371

**Published:** 2019-09-18

**Authors:** Jiayi He, Jianling Zhang, Xuesong Li, Hong Wang, Cui Feng, Feng Fang, Sainan Shu

**Affiliations:** ^1^Department of Pediatrics, Tongji Hospital of Tongji Medical College, Huazhong University of Science and Technology, Wuhan, China; ^2^Departments of Internal Medicine and Genetic Diagnosis Center, Tongji Hospital of Tongji Medical College, Huazhong University of Science and Technology, Wuhan, China; ^3^Department of Radiology, Tongji Hospital of Tongji Medical College, Huazhong University of Science and Technology, Wuhan, China

**Keywords:** citrin deficiency, failure to thrive and dyslipidemia caused by citrin deficiency, SLC25A13, alpha-fetoprotein, hepatocellular carcinoma

## Abstract

Citrin deficiency initially presents as neonatal intrahepatic cholestasis (NICCD) and often resolves within first year of infancy. Failure to thrive and dyslipidemia caused by citrin deficiency (FTTDCD) has been recently proposed as a novel post-NICCD phenotype and its clinical features are still being established. Herein, we encountered a 2-year-old girl who was hospitalized for intermittent fever lasting 10 days. Besides pneumonia, we observed an NICCD-like phenotype with the presence of liver dysfunction, dyslipidemia, aminoacidemia, organic academia, and extremely high levels of alpha-fetoprotein (AFP). Genetic testing confirmed the diagnosis of citrin deficiency and, liver histology revealed she had already developed cirrhosis. Although, improvement of biochemical parameters and liver histology were observed after treatment that included dietary restrictions and symptomatic treatments, AFP levels remained elevated (>400 ng/ml) during a 3-year follow-up period. Moreover, liver magnetic resonance imaging (MRI) examination performed on the patient at age 5 revealed the development of multiple liver nodules with diffusion restriction on diffusion-weighted imaging (DWI). These observations highly indicate the possibility of hepatocellular carcinoma (HCC). Thus, this case reveals that an NICCD-like phenotype complicated with cirrhosis can exist during FTTDCD stage without any prior signs. It also emphasizes the necessity of monitoring AFP levels during follow-up for citrin deficiency patients with persistently high AFP level after treatment as FTTDCD may progress to HCC. Individualized treatment strategy for patients with FTTDCD also need to be explored.

## Introduction

Citrin deficiency is an autosomal recessive metabolic disease caused by the pathogenic mutation of SLC25A13 gene (1). The clinical characteristics of citrin deficiency vary with age. It contains three main phenotypes: neonatal intrahepatic cholestasis caused by citrin deficiency (NICCD) in newborns or infants (0–1 year old) (2), failure to thrive and dyslipidemia caused by citrin deficiency (FTTDCD) in older children (1–11 years old) (3), and adult-onset citrullinemia type II (CTLN2) in adolescents and adults (11–79 years old) (4).

The majority of NICCD cases present in the first few months of life with symptoms characterized by cholestasis, hepatosplenomegaly, liver dysfunction, aminoacidemia, and extremely high alpha-fetoprotein (AFP) levels (2). The clinical symptoms of NICCD are often ameliorated within 1 year after birth and infants remain healthy after resolution of the symptoms (5). However, in some cases infants develop end-stage liver disease necessitating liver transplantation or resulting in death in the first year of life (6, 7), while in other cases, they may develop FTTDCD or CTLN2 several years later (3, 8). FTTDCD has been recently proposed as a novel intermediate phenotype, which manifests as growth restriction and dyslipidemia (3). It can also present as other non-specific symptoms, such as severe fatigue, anorexia, pallor, drowsiness, abdominal discomfort, and headache (8). Currently, the clinical features of FTTDCD remain largely unclear (9).

Many studies have reported an association between hepatocellular carcinoma (HCC) and CTLN2 (10, 11). Recently, a case was reported where a 6-year-old boy with elevated transaminase levels was diagnosed with citrin deficiency and developed advanced HCC (12). Herein, we report a 2-year-old girl who exhibits a NICCD-like phenotype complicated with cirrhosis at the FTTDCD stage without any prior signs. Additionally, during 3-year follow-up period, she had persistently elevated AFP levels and abnormal nodules on liver imaging, strongly indicating the possibility of HCC.

## Case Report

The patient, a girl who had been full-term at birth via normal delivery weighing 3,000 g and had normal neonatal mass screening results and was breast-fed. During the neonatal period, she had a transient history of neonatal jaundice which was spontaneously resolved. Her growth and development were normal during infancy. She was the third child of healthy non-consanguineous parents of Chinese origin. Her elder brother had prolonged jaundice and eventually died of unexplained liver failure at the age of 1.

**Figure 1:**
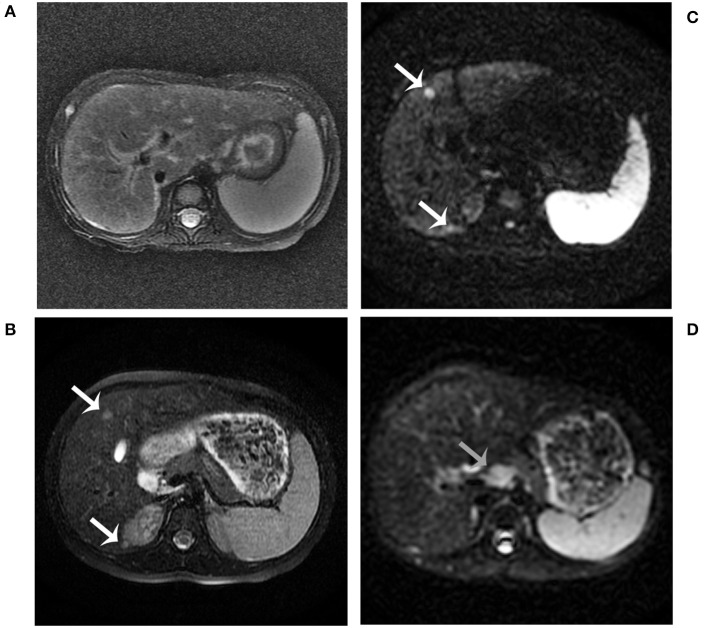
Abdominal MRI findings. MRI examination was performed in the patient at age 2 years. It showed hepatosplenomegaly, portal tract edema, and a slightly low signal intensity of the liver on T2-weighted imaging **(A)**. MRI combined with diffusion-weighted imaging (DWI) examination was performed in the patient at age 5 years **(B–D)**. The nodules showed a slightly high signal intensity on T2-weighted imaging (**B**, white arrows). The nodules showed restricted diffusion on DWI (**C**, white arrows). Enlarged retroperitoneal lymph nodes showed restricted diffusion on DWI (**D**, gray arrows).

**Figure 2:**
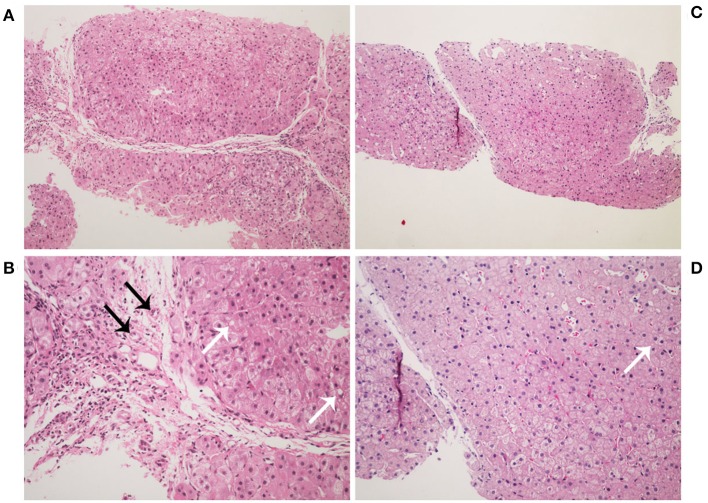
Histopathology images of liver stained by hematoxylin-eosin. Liver histology in the patient at age 2 years **(A,B)**. It showed a typical picture of cirrhosis with regenerative nodules of hepatocytes separated by fibrous septa (**A**, ×100). It also shows hydropic degeneration, microvesicular fatty droplets (white arrows), bile duct proliferation (black arrows), and inflammatory cells infiltration (**B**, ×200). Liver histology in the patient at age 4 years **(C,D)**. It showed lower hydropic degeneration rate and fewer inflammatory cells infiltration.

At 2 years of age, she was referred to our hospital for a 10-day history of intermittent fever. Physical examination revealed fever (temperature to 38.8°C). Patient's weight was 13 kg (75th−90th centile) and height was 85 cm (10th−25th centile). Moist rale of lungs was heard. The liver and spleen were palpable 3.5 and 3 cm under the rib cage, respectively. Yellowing of the skin and sclera and other specific signs were not observed. Laboratory tests on admission are shown in Table 1. Routine blood test showed a slight decrease in the counts of erythrocyte, neutrophil, and platelet. In addition, mildly raised C-reactive protein levels and abnormal result of the chest X-ray indicated the existence of pneumonia. Unexpectedly, serum levels of alanine transaminase, aspartate transaminase, alkaline phosphatase, gamma-glutamyl transpeptidase, total bilirubin, direct bilirubin, total bile acid, and triglyceride were elevated. However, serum level of high-density lipoprotein was decreased. This indicated the existence of cholestasis and dyslipidemia. Based on these clinical and biochemical findings, diagnostic laboratory tests for cholestatic liver disease were performed. Tests for infectious causes including hepatitis A, B, and C viruses were negative, as well as for IgM antibodies to toxoplasma, rubella virus, cytomegalovirus, herpes simplex virus, Epstein-Barr virus, and human parvovirus B19. In addition, the serum levels of ammonia, pyruvate, lactate, and copper were normal. Notably, serum AFP level was extremely elevated (Table 1). Upper abdominal ultrasonography showed enlargement of echogenic dots, increased echoes, and uneven distribution within the liver parenchyma together with splenomegaly, which indicated the possibility of cirrhosis. Liver magnetic resonance imaging (MRI) also revealed the same result (Figure 1). At the same time, light microscopy examination of a liver needle biopsy showed cirrhosis, as evidenced by loss of normal hepatic lobular architecture with fibrous septa separating and surrounding and formation of pseudo-lobule. Proliferation of the bile duct and infiltration of inflammatory cells in the portal tracts were observed. Fat droplets were found in the cytoplasm of some hepatocytes (Figure 2). Therefore, a clinical suspected diagnosis of citrin deficiency complicated with cirrhosis was made. Tandem mass spectrometry analysis of serum amino acid revealed slightly elevated levels of methionine and glycine, while the citrulline level was in the normal range. Urinary gas chromatography-mass spectrometry analysis revealed large quantities of 4-hydroxyphenylacetic acid, 4-hydroxyphenyllactic acid, and 4-hydroxyphenylpyruvate. Mutation analysis of the SLC25A13 gene revealed that the patient was homozygous for a known mutation c.852_855delTATG (p.Met285fs), which resulted in a frameshift deletion of 4 nucleotides and a premature truncation of the protein (8). Sequencing homologous regions of her parents and younger brother revealed they were heterozygous for this sequence variation. Thus, the diagnosis of citrin deficiency complicated with cirrhosis was confirmed.

**Table 1:** Results of blood examinations.

**Biochemical examination**	**Normal range**	**Age**		
		2 years^*^	**4 years**	**5 years**
Hemoglobin (g/L)	115–150	95	117	ND
Neutrophils (10^9/*L*^)	1.80–6.30	1.04	3.52	ND
Platelet (10^9/*L*^)	125–350	98	170	ND
C-reactive protein (mg/L)	0–8	12.3	ND	ND
Alanine aminotransferase (U/L)	4–33	126	20	25
Aspartate aminotransferase (U/L)	4–32	289	41	38
Albumin (g/L)	38–54	36.9	42.4	48.6
Total bilirubin (μmol/L)	3.4–20.5	37.6	2.1	5.9
Conjugated bilirubin (μmol/L)	0–6.84	27	0.7	2.7
Alkaline phosphatase (U/L)	1–281	531	295	290
γ-Glutamyl transpeptidase (U/L)	6–42	367	144	68
Total bile acid (μmol/L)	1–10	152.5	21	10.9
Total cholesterol (mmol/L)	<5.18	3.62	2.50	2.90
Triglyceride (mmol/L)	0.05–1.70	2.68	1.96	1.84
High density lipoprotein (mmol/L)	1.10–1.90	0.35	1.08	1.12
Ammonia (μmol/L)	11–51	39	51	ND
Pyruvate (μmol/L)	20–100	<30	<30	ND
Lactate (mmol/L)	0.50–2.20	1.06	0.95	ND
Citrulline (μmol/L)	3–43	36	23	ND
Methionine (μmol/L)	3–60	318	37	ND
Glycine (μmol/L)	120–550	637	260	ND
Alpha-fetoprotein (ng/ml)	0.6–7.0	25,883	674	425

* *The first time she referred to our hospital; ND, not done*.

The patient began to receive medical treatment (ursodeoxycholic acid and reduced glutathione) combined with high-protein, high-fat, and low-carbohydrate diets when citrin deficiency was suspected, and then was discharged when the symptoms improved. During the follow-up period, except for unique food preferences and overweight, she did not show any other symptoms. During this time, biochemical parameters gradually improved and returned to normal value, except for AFP levels (674 ng/ml). Liver biopsy was performed when she was 4 years old. It showed lower hydropic degeneration rate and fewer inflammatory cells infiltration, which indicated the improvement of liver histology. However, we didn't observe the portal area in this specimen (Figure 2). Considering that a persistently elevated AFP level in the context of cirrhosis was a risk factor of HCC, we continued to follow up closely. The patient underwent liver MRI examination at 5 years old. It showed abnormal multiple nodules on T2-weighted imaging, which were new as compared with the previous result. In addition, it also revealed liver cirrhosis, splenomegaly, and enlarged mesenteric and retroperitoneal lymph nodes. What's more, these nodules and lymph nodes had diffusion restriction on diffusion-weighted imaging (DWI) (Figure 1). Though the blood biochemical indicators were normal during the follow-up, persistently elevated AFP levels (>400 ng/ml for 3 years) in the context of cirrhosis along with the MRI result, made us consider the possibility of HCC.

## Discussion

Citrin functions as a calcium-stimulated aspartate-glutamate carrier in the liver (13). It exports aspartate from the mitochondria to the cytosol for urea, protein, and nucleotide synthesis in hepatocytes. In addition, it also functions as a component of malate-aspartate shuttle, which not only maintains a balance in the relationship between nicotinamide adenine dinucleotide dehydrogenase (NADH) and nicotinamide adenine dinucleotide (NAD+), but also participates in glycolysis and gluconeogenesis (8). Given the important and multiple roles of citrin, its deficiency can manifest in a wide range of symptoms (14).

Currently, the pathophysiology and clinical manifestations of FTTDCD remains largely unknown (9, 14). The case presented here showed an NICCD-like phenotype and can also exist during FTTDCD stage. For this patient, the clinical evidences included hepatosplenomegaly, liver dysfunction, cholestasis, dyslipidemia, aminoacidemia, and extremely high AFP levels which were consistent with the manifestations of NICCD. Genetic testing for SLC25A13 mutation supported the diagnosis of citrin deficiency. On the other hand, it was not until she was hospitalized for fever caused by pneumonia that citrin deficiency was found. Unfortunately, cirrhosis already existed at that moment. It is noted that early signs may be concealed or dismissed in some citrin deficiency patients. This patient had a transient history of neonatal jaundice and her younger brother had prolonged jaundice and died of liver failure. An NICCD case reported by Zhang et al. presented an apparently healthy 8-month-old infant with an acute liver failure precipitated by infection (7). A retrospective study showed late referral was associated with poor prognosis in NICCD; and suggested that any infant of East Asian origin with neonatal cholestasis should be screened for any clues of NICCD (15). Therefore, early detection is necessary and better screen strategies are needed.

This case was unique in that her AFP levels remained at a moderate elevated level (>400 ng/ml) in the FTTDCD stage with normal biochemical parameters during a 3-year follow-up period. The AFP levels dramatically decreased in the initial phase of the treatment. However, it did not decrease to a normal range when the symptoms resolved as was previously reported (16). AFP concentration was also normal in individuals with CTLN2 except that some individuals have CTLN2 associated with HCC (17). Many case reports also pointed to an epidemiological connection between HCC and CTLN2 (10, 11). In addition, the presence of persistently elevated AFP levels in patients with cirrhosis was a risk factor for HCC (18). Therefore, we suggested MRI examination and the abnormal findings of liver imaging indicated the liver lesions may progress to HCC. Several potential mechanisms linking citrin deficiency to hepatocarcinogenesis have been proposed. Preneoplastic changes can result from alterations in gene expression conferred by various factors including loss of heterozygosity (LOH), which can amplify oncogenes or inactivate tumor suppressor genes (19). The odds ratio for carriers of SLC25A13 mutations among non-viral HCC patients vs. the general population was 6.6, suggesting that patients with SLC25A13 gene mutation may be more likely to develop HCC (20). In addition, patients with citrin deficiency have a downregulation of PPARα, which leads to fatty acids not being effectively oxidized and thus accumulating in the liver (21). Augmented oxidative stress was observed in children with citrin deficiency even during the silent period (22). And Fatty acids and oxidative stress may contribute to persistent liver damage and hepatocarcinogenesis in citrin deficiency (23, 24). In sum, merely monitoring of liver function tests is insufficient in the follow-up of citrin deficiency patients and serum AFP level should be also included. In addition, when patients with citrin deficiency have a persistently elevated AFP level, the possibility of HCC should be considered.

Current treatment strategies for FTTDCD include diet therapy (low carbohydrate and high protein and fat) and supplements (e.g., administration of sodium pyruvate and/or MCT) (25). A case reported by Otsuka et al. (26) showed MCT therapy can prevent the onset of hypoglycemia during the so-called apparently healthy period. In addition, it is speculated that long-term MCT supplementation may prevent a complication of HCC by suppressing the Wnt/β-catenin pathway (27). An hCitrin-mRNA-based therapy has recently been reported to have a significant therapeutic effect in a murine model of citrin deficiency, and could be a promising therapy for citrin deficiency (28). For this patient, she resolved except for high AFP levels after diet therapy. It is uncertain whether she would benefit from MCT treatment. Therefore, a cohort study of MCT supplementation on citrin deficiency patients during the apparently healthy period need to be started and individualized treatment strategy for patients with FTTDCD need to be explored.

The findings in this case report revealed that an NICCD-like phenotype complicated with cirrhosis can present without any prior signs at FTTDCD stage; if early signs are concealed or dismissed this could result in late referral and may finally lead to poor outcomes. Early detection and intervention are very important. Dynamic monitoring of AFP levels should be included in the follow-up of citrin deficiency patients with persistently high AFP level after treatment as FTTDCD may progress to HCC. Dietary therapy was partially effective in our case since the patient's liver lesions still progress. Individualized treatment strategy for patients with FTTDCD need to be further explored.

## Concluding Remarks

The case we report here reveals that an NICCD-like phenotype complicated with cirrhosis can exist during FTTDCD stage without any prior signs. It also emphasizes the necessity of monitoring AFP levels during follow-up for citrin deficiency patients with persistently high AFP level after treatment as FTTDCD may progress to HCC.

## Data Availability

All datasets analyzed for this study are included in the manuscript and the supplementary files.

## Ethics Statement

This study was conducted in accordance with Human Ethics Committee of Huazhong University of Science and Technology. The patient's parents are completely informed about all the tests both verbally and in written format. We also obtained written and informed parental consent for publication of this case report.

## Author Contributions

JH, JZ, and SS conceptualized and designed the study, wrote, reviewed, and revised the manuscript. XL, CF, and FF did the follow-up study, collected and interpreted the data, and managed the patient. HW did the genetic test. All authors approved the final manuscript as submitted and agree to be accountable for all aspects of the work.

### Conflict of Interest Statement

The authors declare that the research was conducted in the absence of any commercial or financial relationships that could be construed as a potential conflict of interest.
